# The Effect of the COVID-19 Vaccine on the Menstrual Cycle Among Reproductive-Aged Females in Saudi Arabia

**DOI:** 10.7759/cureus.32473

**Published:** 2022-12-13

**Authors:** Asma M Alahmadi, Amal H Aljohani, Ruba A Fadhloun, Areej S Almohammadi, Dorar F Alharbi, Lama S Alrefai

**Affiliations:** 1 Obstetrics and Gynaecology, Taibah University, Medina, SAU; 2 Family Medicine, Yanbu General Hospital, Yanbu, SAU; 3 College of Medicine, Taibah University, Medina, SAU; 4 Pharmacy, Alnahhas Trading Company, Medina, SAU

**Keywords:** saudi arabia, reproductive-aged females, covid-19 vaccine, menstrual changes, menstrual cycle

## Abstract

Background: A global concern about a possible association between COVID-19 vaccines and menstrual disturbance has been raised. Moreover, women who have experienced menstrual changes are worried about the length of the side effects and are hesitant to receive booster doses. Therefore, the aim of this study is to evaluate the impact of the COVID-19 vaccine on all features of the menstrual cycle, including cycle length, amount of bleeding, and pain.

Methodology: We retrospectively analyzed menstrual cycles following at least two doses of COVID-19 vaccines; the cycle changes within the individual pre-vaccination and post-vaccination were compared. All reproductive-aged females from 18 to 45 years who fit the inclusion criteria were included in the study and categorized into five sub-categories based on age to investigate whether certain age groups were most affected. The data were collected through a well-structured self-administered questionnaire. Participants obtained their vaccination information (date, type of vaccine) from Tawakkalna, the official COVID-19 application in the Kingdom of Saudi Arabia. IBM Corp. Released 2019. IBM SPSS Statistics for Windows, Version 26.0. Armonk, NY: IBM Corp was performed in data entry and statistical analysis. Variables were described as frequency and percentage, as all were categorical. To investigate the association between menstrual changes and its possible associated factors, we used the Chi-square test, and the statistical significance was determined at p<0.05.

Results: The online questionnaire received responses from a total of 1092 reproductive females. However, out of which, 419 were not fitting into the inclusion criteria. Thus, a total of 673 females were included in the final report. Overall, the changes in the menstrual cycles after both COVID-19 vaccine doses were observed among 46.7%, mainly more menstrual pain in 22.9% following the first dose compared with 21.4% after the second. Menstrual changes were observed among almost two-thirds of women in the age groups 18-22 years (65.2%) and 38-45 years (65.4%) compared with only 43.5% of those in the age group 23-27 years, p<0.001. The Moderna vaccine was associated with the highest rate of menstrual changes (65.4%), whereas Oxford-AstraZeneca was associated with the lowest rate (44.9%), p=0.040. The duration of changes in the cycles after the COVID-19 vaccine (one dose or both) was less than one month among 42.5% of females, whereas it was three months or more among 27.1%.

Conclusion: The COVID-19 vaccination is associated with a minor and transient change in the menstrual cycle, resulting mainly more menstrual pain and increased bleeding.

## Introduction

Following the first emergence that was reported in late December 2019 in China, The novel coronavirus disease (COVID-19) spread rapidly around the world and was designated a global pandemic by the World Health Organization (WHO) on March 11, 2020 [1]. It is an infectious disease caused by SARS-CoV-2 and has killed more than six million people worldwide, making it one of the most significant global health crises since the 1918 influenza pandemic [2]. Vaccination is the most effective method of preventing the spread of infectious diseases and decreasing morbidity, mortality, and hospitalization [3]. Since December 2020, many COVID-19 vaccines have been developed and approved for use.

Currently, in Saudi Arabia, there are three vaccines approved for use against SARS-CoV-2. On December 10, 2020, the Pfizer-BioNTech vaccine was the first to be approved for use in Saudi Arabia. On February 18, 2021, the chimpanzee adenovirus vector vaccine ChAdOx1 by Oxford-AstraZeneca was the second vaccine to be approved for use in Saudi Arabia. Followed by Moderna, which was approved on July 9, 2021. AstraZeneca was developed using a replication-deficient simian adenovirus vector, while Pfizer and Moderna were developed using mRNA technology [4]. In Saudi Arabia, until 15^th^ November 2022, around 67 million vaccine doses have been given, and 15 million of the population received the booster dose [5]. The safety and efficacy of vaccines have been approved through extensive clinical trials. Common side effects following vaccination, including high temperature, fatigue, headache, muscle pain, and pain at the injection site, have been reported by many people. However, side effects were minor and resolved within days [6]. Furthermore, unexpected menstruation changes are not mentioned as one of the symptoms, but we are increasingly approached by women of all ages who have had these changes after the COVID-19 vaccine. Menstrual irregularities are an extremely common complaint in gynecology clinics. The majority of affected women will strongly impact their quality of life. The concern about a possible association between vaccination against COVID-19 and menstrual disturbance has been raised considering the post-vaccination symptoms [7]. In addition, young women's hesitancy to get vaccinated is largely due to inaccurate claims that the COVID-19 vaccine could affect future fertility [7].

The International Federation of Gynecology and Obstetrics (FIGO) defined standardized parameters for typical menstruation regarding menstrual frequency, duration, regularity, and quantity, and deviation from these may constitute abnormal uterine bleeding [8]. Upon review of the literature, there were few studies conducted recently to investigate this association. A retrospective cohort study was conducted in the United States (US) from October 2020 to September 2020 on women 18-45 years of age (including 3,959 women [vaccinated: 2,403 and unvaccinated: 1,556]) with normal cycle lengths for three successive cycles before the first vaccine dose and three consecutive cycles afterward [9]. Those women were compared with unvaccinated women with six cycles over a similar period. The women who received the vaccine had their cycle lengths changed by less than one day. However, the unvaccinated women noticed no significant changes. Another retrospective case-control study was conducted in the United Kingdom (UK) with 4,989 participants between the ages 28 and 43 years who were pre-menopausal and had been vaccinated [10]. Four months afterwards their first COVID-19 vaccination, 80% of women reported no menstrual cycle changes. Only 20% reported a menstrual disturbance. In a recent preprint of a US web-based survey recruiting only vaccinated participants through Twitter and other social media platforms [11]. After vaccination, 42% of women with regular menstrual cycles reported bleeding more than usual, whereas 44% reported no change. Another study conducted in Norway included 5688 women aged 18-30 years [12]. They used the first six weeks post-vaccination as the exposure period to estimate the relative risk of menstrual changes according to vaccination in a self-administered case series design. After vaccination, they found a significant increase in menstrual changes, In particular short intervals between menstruations, heavier bleeding, and longer duration. When comparing the exposed period with an unexposed period, they found the relative risk of more heavy bleeding than usual with the first dose vaccine was 1.90 (95% CI: 1.69-2.13), whereas 1.84 (1.66-2.03) for the second dose.

In Saudi Arabia, there was a study conducted in October 2021 investigating Pfizer BioNTech and Oxford-AstraZeneca post-vaccination side effects in general [13]. Menstrual disturbances were reported in an open question section, abnormal menstrual cycle (delaying/increasing /hemorrhages or pain): 0.98% of Pfizer BioNTech and 0.68% (7/1028) of Oxford-AstraZeneca vaccines, in a cross-sectional study of women living in the Middle East and North Africa (MENA) from July 2021 to August 2021 [14]. A total of 2269 women with a mean age of 34.3 ± 8.5 years were included in their study. Approximately 66.3% of women reported menstrual cramps after vaccination, 46.7% of which occurred after the first vaccination. However, 93.6% of women's symptoms disappeared within two months. Vaccination type had no significant effect on abnormal incidence (p > 0.05). In this study, our primary objective is to evaluate the impact of the COVID-19 vaccine on all features of the menstrual cycle, including cycle length (in days), duration (in days), amount, and pain. Furthermore, as a sub-analysis, we aim to categorize our sample (reproductive-aged females from 18 to 45 years) into five categories based on age to investigate whether certain age groups will be most affected.

## Materials and methods

Over 12 months, we conducted a retrospective cohort study (From January 2021 to January 2022). The data were collected through a well-structured self-administered questionnaire that was sent to the participants via various social media platforms: WhatsApp, Twitter, Snapchat, and Instagram. The consent was secured before starting the survey participants were fully informed about the objectives and purpose of the questionnaire. Furthermore, the assurance of confidentiality before questionnaire filling was given. The questionnaire comprises demographic information, including age, marital status, residence, and level of education. Moreover, questions addressing menstrual cycle features (e.g., length, duration, amount, and pain) regarding pre-and post-COVID-19 vaccines.

The inclusion criteria were all reproductive-aged women between 18 and 45 years with a normal menstrual cycle (cycle length between 21-35 days and menses duration less than eight days) and those who received two doses of COVID-19 vaccines regardless of vaccine type. However, the following women were excluded from this study: women known to have polycystic ovarian syndrome (PCOS), thyroid disorders, hyperprolactinemia, were pregnant, used hormonal birth control (i.e., birth control pills, injections, or implants), and hormone-containing intrauterine devices (IUDs). These women were asked about the inclusion and exclusion criteria before filling out the survey questionnaire. Participants obtained the vaccination information (date, type of vaccine) from Tawakkalna, the official COVID-19 application in the Kingdom of Saudi Arabia; participants can review or download the health passport where they found the type and date of vaccination.

Before starting the study, ethical approval was obtained from the Research Ethics Committee, College of Medicine at Taibah University, Medina, Saudi Arabia (Reference Number: IORG0008716 -IRB00010413). Our primary outcome is to investigate the impact of two doses of the COVID-19 vaccination on the menstrual cycle with respect to cycle length, duration of menses, frequency, amount, and intermenstrual bleeding. The cycle changes within the individual pre-vaccination and post-vaccination were compared. We compared the last three regular pre-vaccination cycles with the first, second, and third cycles following vaccination. Our secondary outcomes were to investigate the duration of cycle changes and the effect of the type of vaccine on the menstrual cycle. Moreover, we divided the sample into five main categories based on female age as follows: 18-22, 23-27, 28-32, 33-37, and 38-45 to investigate whether certain age groups were most affected. IBM Corp. Released 2019. IBM SPSS Statistics for Windows, Version 26.0. Armonk, NY: IBM Corp was used for data entry and statistical analysis. Variables were described as frequency and percentage, as all were categorical. To investigate the association between menstrual changes and its possible associated factors, we used the Chi-square test, and the statistical significance was determined at p<0.05.

## Results

Demographic characteristics

A total of 1092 reproductive females responded to the online questionnaire. Of them, 419 were excluded as they included one or more exclusion criteria. Thus, a total of 673 females were included in the final report. Demographic characteristics are presented in Table 1. The age of 42% ranged between 23 and 27 years, whereas 7.7% ranged between 38 and 45 years. Singles represented 75% females, and 54.8% lived in Al-Madinah city. Almost two-thirds (67.8%) held a Bachelor's degree.

**Table 1:** Demographic characteristics of the participants (n=673).

		Frequency	Percentage
Age (years)	18-22	224	33.3
	23-27	283	42.0
	28-32	80	11.9
	33-37	34	5.1
	38-45	52	7.7
Marital status	Single	505	75.0
	Married	154	22.9
	Divorced/widowed	14	2.1
Residence	Al-Madinah	369	54.8
	Others	304	45.2
Educational level	< high school	11	1.6
	High school/equivalent	168	25.0
	Bachelor's degree	456	67.8
	Postgraduate degree	38	5.6

COVID-19 vaccine uptake and menstrual disturbances

The majority of eligible participants (76.5%) have received three vaccine doses against COVID-19. The Pfizer-BioNTech vaccine was the most commonly reported either for the first dose (75.8%) or second (74.3%), followed by Oxford-AstraZeneca (23% and 21.8% for the first and second dose, respectively). Menstrual changes were observed among 45.9% of patients after the first dose, primarily increased menstrual pain (22.9%) and increased menstrual bleeding (14.3%), whereas menstrual changes were observed among 42.8% of patients after the second dose; in general increased menstrual pain (21.4%) and intermenstrual bleeding (12.5%). The duration between the first dose and the booster was three months or more among the majority of participants (60.3%) (Table 2).

**Table 2:** History of COVID-19 vaccine uptake and menstrual disturbance among the participants (n=673). *Not mutually exclusive (sum exceeded 100%).

		Frequency	Percentage
Number of doses	Two	158	23.5
	Three	515	76.5
First vaccine dose	Pfizer-BioNTech	510	75.8
	Oxford-AstraZeneca	155	23.0
	Moderna	8	1.2
Menstrual changes after the first dose	No	364	54.1
	Yes*	309	45.9
	Increase in menstrual cycle length	82	12.2
	Decrease in menstrual cycle length	94	14.0
	More pain	154	22.9
	Increase in amount	96	14.3
	Decrease in amount	29	4.3
	Intermenstrual bleeding	91	13.5
Duration between the first dose and the booster	Less than one month	109	16.2
	1-2 months	158	23.5
	≥3 months	406	60.3
Second vaccine dose	Pfizer-BioNTech	500	74.3
	Oxford-AstraZeneca	147	21.8
	Moderna	26	3.9
Menstrual changes after the second dose	No	385	57.2
	Yes*	288	42.8
	Increase in menstrual cycle length	79	11.7
	Decrease in menstrual cycle length	72	10.7
	More pain	144	21.4
	Increase in amount	81	12.0
	Decrease in amount	27	4.0
	Intermenstrual bleeding	84	12.5
Duration of changes in the cycles after COVID-19 vaccine (one dose or both doses) (n=303)	Less than one month	129	42.5
	1-2 months	92	30.4
	≥3 months	82	27.1

**Figure 1:**
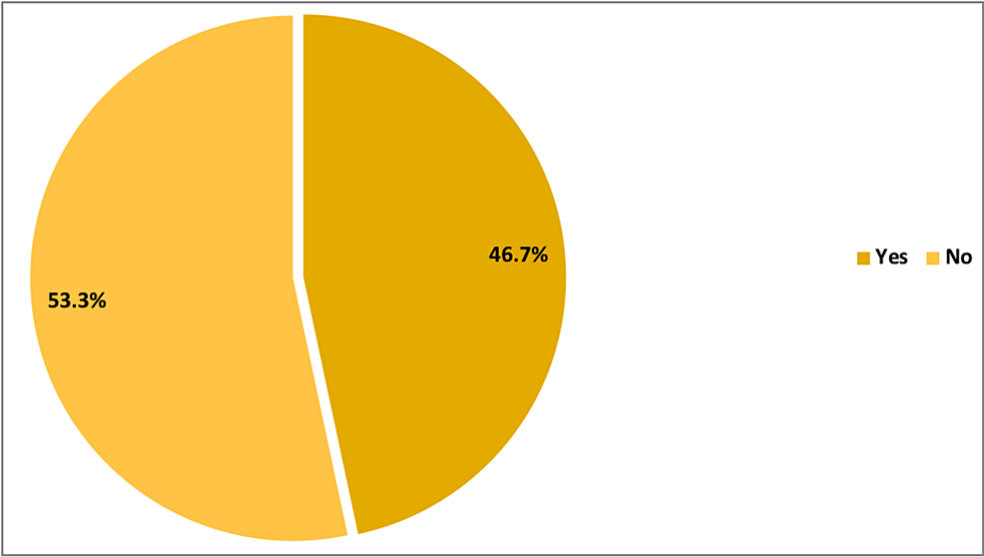
Rate of change in menstrual cycles after one dose or both COVID-19 vaccines.

Overall, menstrual cycle changes after one or both of the COVID-19 vaccines were observed among 46.7% of the participants, as illustrated in Figure 1. The duration of changes in the cycles after the COVID-19 vaccine (one dose or both) was less than one month among 42.5% of females, whereas it was three months or more among 27.1% of them (Table 2).

Post-COVID-19 vaccination menstrual changes were observed among almost two-thirds of women in the age groups 18-22 years (65.2%) and 38-45 years (65.4%) compared to only 43.5% of those in the age group 23-27 years, p<0.001. In addition, menstrual changes were observed among 63.1% of women with high school/equivalent qualifications compared to only 42.1% of postgraduates, p=0.021. Furthermore, women who have had two doses of the vaccine were more likely to report menstrual changes than those who have had three doses (61.4% vs. 50.9%), p=0.020. Regarding the second type of vaccine, the Moderna vaccine was associated with the highest rate of menstrual changes (65.4%), whereas Oxford-AstraZeneca was associated with the lowest rate (44.9%), p=0.040 (Table 3).

**Table 3:** Factors associated with post-COVID-19 vaccination menstrual changes among the participants. * P<0.05. P-value was determined by the Chi-square test.

		Post-COVID-19 vaccination menstrual changes		p-value*
		No n=314 N (%)	Yes n=359 N (%)	
Age (years)	18-22 (n=224)	78 (34.8)	146 (65.2)	<0.001*
	23-27 (n=283)	160 (56.5)	123 (43.5)	
	28-32 (n=80)	44 (55.0)	36 (45.0)	
	33-37 (n=34)	14 (41.2)	20 (58.8)	
	38-45 (n=52)	18 (34.6)	34 (65.4)	
Marital status	Single (n=505)	240 (47.5)	265 (52.5)	0.660
	Married (n=154)	67 (43.5)	87 (56.5)	
	Divorced/widowed (n=14)	7 (50.0)	7 (50.0)	
Residence	Al-Madinah (n=369)	168 (45.5)	201 (54.5)	0.518
	Others (n=304)	146 (48.0)	158 (52.0)	
Educational level		6 (54.5)	5 (45.5)	0.021*
	High school/equivalent (n=168)	62 (36.9)	106 (63.1)	
	Bachelor's degree (n=456)	224 (49.1)	232 (50.9)	
	Postgraduate degree (n=38)	22 (57.9)	16 (42.1)	
Number of COVID-19 vaccine doses	Two (n=158)	61 (38.6)	97 (61.4)	0.020*
	Three (n=515)	253 (49.1)	262 (50.9)	
First vaccine dose	Pfizer-BioNTech (n=510)	225 (44.1)	285 (55.9)	0.063
	Oxford-AstraZeneca (n=155)	85 (54.8)	70 (45.2)	
	Moderna (n=8)	4 (50.0)	4 (50.0)	
Second vaccine dose	Pfizer-BioNTech (n=500)	224 (44.8)	276 (55.2)	0.040*
	Oxford-AstraZeneca (n=147)	81 (55.1)	66 (44.9)	
	Moderna (n=26)	9 (34.6)	17 (65.4)	
Duration between the first dose and the booster	Less than one month (n=109)	49 (45.0)	60 (55.0)	0.571
	1-2 months (n=158)	69 (43.7)	89 (56.3)	
	≥3 months (n=406)	196 (48.3)	210 (51.7)	

Feelings regarding post-COVID-19 vaccination menstrual changes

**Figure 2:**
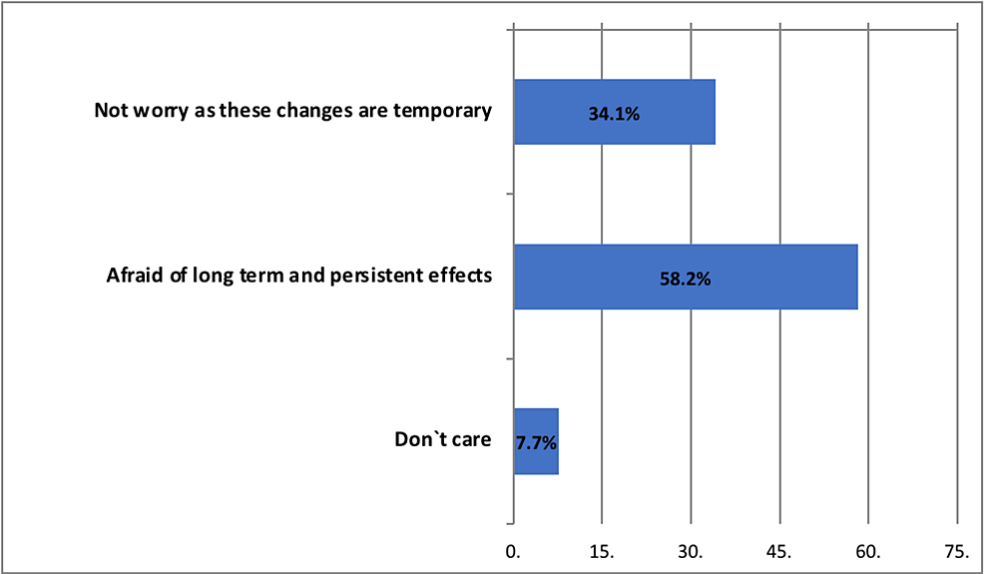
Participants` feelings regarding post-COVID-19 vaccination menstrual irregularities (n=311).

More than half (58.2%) of the participants with post-COVID-19 vaccination menstrual changes were worried regarding long-term and persistent effects, whereas 34.1% were not worried and considered these changes temporary (Figure 2).

Dealing with post-covid-19 vaccination menstrual changes

**Figure 3:**
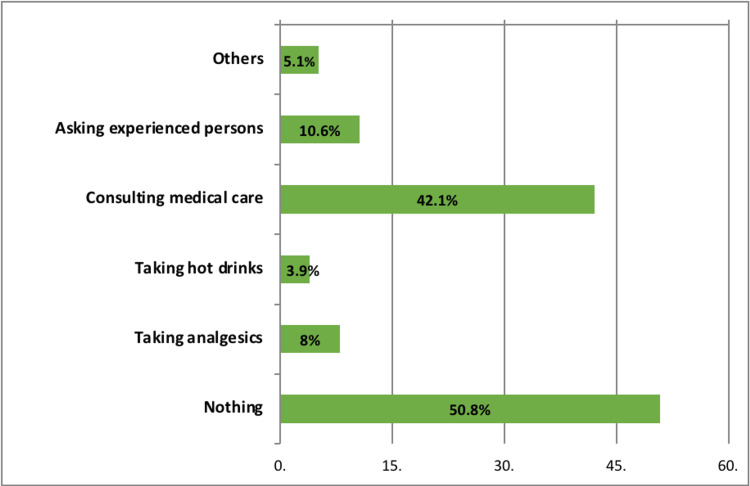
Dealing of the participants with post-vaccination menstrual irregularities (n=311).

More than half of the women with post-COVID-19 vaccination menstrual changes did nothing, whereas 42.1% consulted medical care, and 10.6% asked for help from experienced persons (Figure 3).

## Discussion

In our study, we retrospectively assessed a total of 673 regular menstrual cycles that met our inclusion criteria for at least three months before COVID-19 vaccination. We compared them with three post-vaccination cycles to examine the impact on the menstrual cycle, including cycle length, bleeding volume, menstrual pain, and intermenstrual bleeding. A total of 46.9% of participants were found to have experienced menstrual changes following one or both vaccines, a particularly high rate after the first dose of the COVID-19 vaccine. However, approximately 42.5% of menstrual changes in our sample are transient and disappear within a month or less. This result is consistent with previous studies that included a total of 2269 women, of whom (46.7%) reported menstrual changes, and 86.8% of affected women experienced symptom relief within one month [14]. Abnormal uterine bleeding accompanied by changes in cycle length or duration, changes in bleeding volume, or other cycle characteristics such as menstrual pain prompted 42% of affected women to see a doctor. Our study shows that in 21%-22% of responders, an increase in menstrual pain after the first and second doses was the most common post-vaccination complaint, followed by heavier-than-usual cycles. Our findings are consistent with a preprint of a recent web-based US survey that recruited vaccinated participants via Twitter and other social media platforms and reported that 42% of people with normal menstrual cycles bled more than usual [11]. Another study in Norway, involving 5,688 women between the ages of 18 and 30, found a significant increase in irregular menstruation after vaccination, especially when bleeding was heavier than usual [12].

According to our data, both the first and second doses of the COVID-19 vaccine alter cycle length, with some participants reporting an increase in cycle length, whereas others reporting a decrease in the length of their previous normal and routine cycles. However, we should specify the number of days which was considered one of the limitations of our study. Regarding the vaccine type and its effect on menstrual changes, we found Moderna is associated with the highest rate of menstrual cycle changes for the second dose with a statistically significant value. This contrasts with a previous study that showed vaccine type had no influence on the incidence of menstrual changes. A regular menstrual cycle was 21 to 35 days with 4 to 7 days of bleeding and regulated by the complex hypothalamic-pituitary-gonadal axis. Many factors, such as emotional stress and weight loss, can affect the hypothalamus and, thus, the axis, which led to changes in menstrual cycle characteristics [15].

The finding is difficult to interpret and does not entirely correlate with the COVID-19 vaccine because of known age-related changes in the menstrual cycle, especially after 40 years in pre-menopausal women. However, the exact mechanism remains unclear due to the stress of the COVID-19 pandemic and the potential mechanism that new vaccines may play. Nevertheless, many vaccines have been previously investigated and linked to immune-medicated thrombocytopenia, for example, measles-mumps-rubella (MMR), hepatitis A and B, diphtheria-tetanus-acellular pertussis (DTaP), and varicella [16]. We believe that our study has many strengths: First, it is considered a pioneer to conduct a study on the impact of COVID-19, a sensitive and concerning subject in our community, that addresses all menstrual cycle features, in contrast to another study that addressed the general side effects of COVID-19 vaccines. Second, our data source is the official COVID-19 application in Saudi Arabia, Tawakkalna, where participants can obtain accurate information regarding vaccination. Third, concerning the inclusion criteria, we recruited a cohort with previously regular menstrual cycles to exclude any factors that could have a significant impact on the menstrual cycle and to definitively identify the association between the COVID-19 vaccine and menstrual cycle variability. However, this study has several limitations. The first limitation is that the sample size in the data collection process may only generalize to some women of reproductive age in the Kingdom of Saudi Arabia since the questionnaire is online and some participants might have attempted the questionnaire randomly. The second limitation is that answering the questions about the dates of the two doses varies from one participant to another, as some are within an average of eight months between doses, whereas others are longer in duration. Having said that, several participants do not even remember the accurate date. In addition to those already mentioned, we suggest several research avenues that may be beneficial. Regardless of these limitations, this study advances our understanding of the impact of two doses of the COVID-19 vaccine on the menstrual cycle. We hope that the current study will stimulate further investigation into this important area and avoid our limitations by making it a face-to-face questionnaire in outpatient clinics and using dose dating from the Ministry of Health application.

## Conclusions

Our findings are reassuring and consistent with previous research on the impact of COVID-19 vaccines on menstrual cycle features. COVID-19 vaccination is associated with minor and transient changes in the menstrual cycle. Women who received at least two doses of the vaccine primarily reported more menstrual pain and increased bleeding, but these changes resolved within a short period amongst most participants. This helps reassure women of reproductive age and provides healthcare professionals with preliminary evidence to advise women seeking medical advice on the impact of COVID-19 on their menstrual cycle and is beneficial in encouraging women to receive booster doses. Further research is required to demonstrate the association and long-term effects of the COVID-19 vaccine on the menstrual cycle.
